# Changes in the gut microbiota composition of healthy young volunteers after administration of *Lacticaseibacillus rhamnosus* LRa05: A placebo-controlled study

**DOI:** 10.3389/fnut.2023.1105694

**Published:** 2023-03-14

**Authors:** Zhonghui Gai, Yao Dong, Fei Xu, Junli Zhang, Yujiao Yang, Yuwen Wang

**Affiliations:** ^1^Department of Research and Development, Wecare-Bio Probiotics (Suzhou) Co., Ltd., Suzhou, China; ^2^College of Food Science and Technology, Henan University of Technology, Zhengzhou, China; ^3^Henan Province Wheat-Flour Staple Food Engineering Technology Research Centre, Zhengzhou, China

**Keywords:** *Lacticaseibacillus rhamnosus*, gut microbiota, probiotic, healthy young volunteers, a placebo-controlled study

## Abstract

The gut microbiota promotes gastrointestinal health in humans; however, the effect of probiotics on the gut microbiota of healthy adults has not been documented clearly. This placebo-controlled study was conducted to assess the effect of *Lacticaseibacillus rhamnosus* LRa05 supplementation on the gut microbiota of healthy adults. The subjects (*N* = 100) were randomized 1:1 to receive (1) maltodextrin (placebo, CTL group) and (2) maltodextrin + strain LRa05 (1 × 10^10^ colony-forming units/day, LRa05 group). The duration of the intervention was 4 weeks, and changes in the gut microbiota from before to after the intervention were investigated using 16S rRNA high-throughput sequencing. In terms of alpha diversity, no significant difference in the composition of the gut microbiota was found between the LRa05 and CTL groups. 16S rRNA sequencing analysis showed that the relative abundance of *Lacticaseibacillus* significantly increased after supplementation with LRa05. Furthermore, a decreasing trend in the abundance of *Sellimonas* and a significant decrease in the salmonella infection pathway were observed in the LRa05 group compared with the CTL group. These findings indicate the potential of LRa05 to colonize the human gut and reduce the abundance of harmful bacteria in the microbiota.

## Introduction

Functionally, microbes in the human gut contribute to various aspects of health by regulating the immune system, fermenting dietary fiber, inhibiting pathogen colonization, and synthesizing vitamins (1). A disturbance of the gut microbiota is associated with the incidence and development of many diseases (2). Probiotic supplementation is a common approach used to alter the gut microbiota and thus improve health. Probiotics are living microorganisms that confer health benefits when consumed in sufficient numbers (3). The mechanisms by which probiotics support the intestinal environment and host health include improving intestinal barrier function through their effects on the epithelial and mucus layers of the gut and producing antimicrobial substances (4).

Probiotics can improve the clinical symptoms of patients with gastrointestinal diseases and regulate the gut microbiota (5, 6). However, few studies have investigated the effects of probiotics on the gut microbiota of healthy people, and these studies have yielded inconsistent conclusions. For example, probiotics have been shown to modulate the gut microbiota and reduce the relative abundance of harmful bacteria in healthy subjects (7–10). However, several clinical studies have shown that probiotic interventions do not cause significant changes in the fecal microbial composition (11–15). Individual differences in susceptibility to probiotics and cross-study differences in probiotic dosage and intervention duration can influence the observed effects of probiotics (16). Therefore, the health benefits of probiotic supplementation should be demonstrated in light of the impact on relevant host phenotypes, especially in healthy participants.

The therapeutic effects of *Lacticaseibacillus rhamnosus* strain LRa05 (hereafter, LRa05) have been described in mouse models of obesity and type 2 diabetes (17, 18). In animal experiments, we demonstrated the safety of LRa05 and its efficacy in regulating the gut microbiota (17). Animal experiments have also shown that LRa05 has gut-localized immunomodulatory effects (18). However, LRa05 has not been reported to have been tested in humans. Accordingly, we focused on assessing the effect of LRa05 on the gut microbiota of healthy adult humans. In this study, healthy young adults were administered 1 × 10^10^ colony-forming units (CFU) of LRa05 daily for 28 days. Each subject’s gut microbiota composition was analyzed before and after probiotic consumption and subjected to assessments of tolerability, safety, and intestinal colonization to determine the effect of LRa05 supplementation on the gut microbiota.

## Materials and methods

### Population recruitment and ethical statement

**FIGURE 1:**
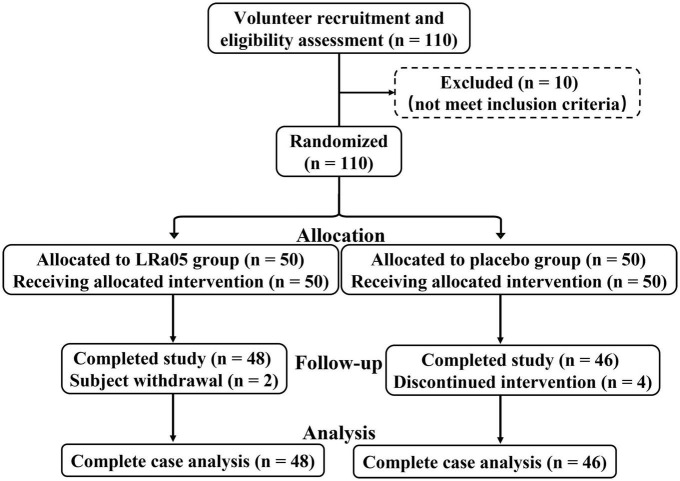
Flowchart of the trial involving healthy volunteers.

The study subjects were healthy volunteers who met the following inclusion criteria: an age between 19 and 45 years, a body mass index (BMI) between 18.5 and 25 kg/m^2^, voluntary acceptance of and adherence to the experimental protocol, and ability to participate in a timely review and follow-up. The exclusion criterion was any autoimmune or other chronic disease. The study was conducted at Henan University of Technology from April 21 to 31 June 2022, and the flow of the trial is shown in Figure 1. Of the 110 initial volunteers, 100 met the above-listed criteria and were included in the study. To exclude the influence of volunteers’ use of other probiotic products on the trial, the probiotic products of all kinds were stopped by communicating with volunteers 2 weeks before enrollment. Prior to formal enrollment, we conducted further inquiry, confirmed that all volunteers meet the conditions before enrollment. The study followed the guidelines of the Declaration of Helsinki, and all procedures involving the human body were approved by the Ethics Committee of Henan University of Technology (No. HautEC202277). Written informed consent was obtained from the study subjects after they had received a detailed explanation of the nature of the study.

### Experimental design

Following a 14 day washout period, the subjects were divided into placebo (CTL) and LRa05 groups. The CTL group received a 2-g supplement of maltodextrin per day, and the LRa05 group received 1.9 g maltodextrin plus 0.1 g LRa05 bacterial powder (1 × 10^10^ CFU) per day. The placebo and probiotic products used in this study were obtained from Wecare Probiotics Co., Ltd. The experimental period was 4 weeks, and the volunteers did not consume products containing probiotics during this period and 2-week washout period. No additional dietary restrictions were imposed, and the subjects maintained their original lifestyle habits during the experimental period.

**FIGURE 2:**
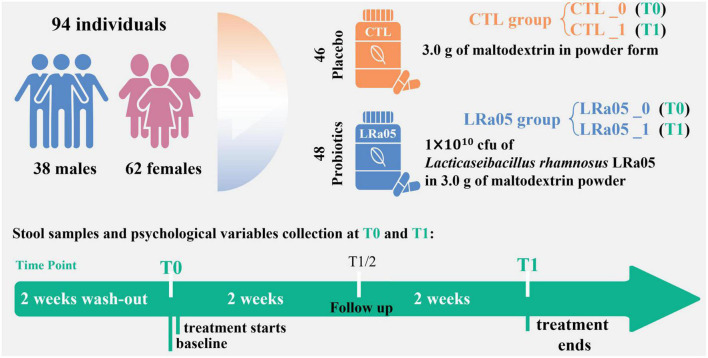
Experimental design of the study. One hundred subjects were enrolled, and 94 subjects completed the trial. We collected fecal samples at baseline (T0) and after the 4-week intervention (T1) and administered questionnaires.

This was a single-blind placebo-controlled trial, meaning that the subjects were unaware of their assigned group. The trial design and process of the study are shown in Figure 2. The study consisted of two visits. During the first visit (T0), each subject’s height, weight, and blood pressure were measured. In the end (T1), each subject’s body fat percentage was measured. We used a questionnaire to evaluate the effect of the placebo or probiotic intervention on the subjects’ defecation. As a questionnaire, Bristol Stool Form Scale (BSFS) is the most widely used evaluation of stool consistency (19), in which types 1 and 2 are considered hard stools (associated with symptoms of constipation); types 3, 4, and 5 are generally considered normal stool forms; and types 6 and 7 are considered abnormally loose or liquid stools (associated with symptoms of diarrhea). To eliminate individual differences, a longitudinal study approach was applied, using samples collected on Day 0 as the control. Fecal samples were collected at T0 and T1 and used to assess the subjects’ gut microbiota (Figure 2).

### Fecal samples, DNA extraction, and sequencing

The subjects were asked to self-collect stool samples, freeze them immediately at −20°C, and bring them to the visit site for storage at −80°C until analysis. DNA was isolated from the samples for testing using the QIAamp DNA kit according to the manufacturer’s instructions. Polymerase chain reaction was used to amplify the V3–V4 variable region of the prokaryotic 16S rRNA gene as previously described (20) using the primers F1 and R2 (5′- CCTACGGGNGGCWGCAG-3′ and 5′-GACTACHVGGGTATCTAATCC-3′). Sequencing was performed on the Miseq platform (Illumina, San Diego, CA, USA) using a 2 × 300 bp paired-end protocol. In preparation for sequencing experiments, a sample that has been sequenced was prepared as a positive control to complete DNA extraction and PCR simultaneously with the sample to be tested.

### Bioinformatic analysis

Methods for bioinformatics analysis of amplicons were adopted from our previous publications (20, 21). Trimmomatic was used to filter low quality sequences (22). Denoising was performed using the UNOISE algorithm, reads were clustered into amplicon sequence variants (ASVs), and classification assignments and construction of ASV tables were performed by using USEARCH software. The sequencing results were analyzed using USEARCH software (version 11.0667),^1^ which produced amplicon sequence variants (ASVs). The 16S rRNA database from the RDP reference training set (version 18) was used as the reference database for sequence annotation.^2^ Community diversity (Shannon and Simpson indices) and richness [Chao1 and abundance-based coverage estimators (ACE)] in the gut microbiota were analyzed at the level of ASVs using the vegan 2.5-7 package (23) on the R platform. The PICRUSt v2.5.0 (Phylogenetic Investigation of Communities by Reconstruction of Unobserved States) pipeline (24) was applied to the 16S rRNA sequencing data, and the imputed relative abundances of Kyoto Encyclopedia of Genes and Genomes (KEGG) pathways in each sample were used to predict alterations in fecal microbiome function using the picrust2_pipeline.py command.

### Statistical analysis

Continuous variable that followed a normal distribution were analyzed using the *t*-test. Continuous variable that did not follow a normal distribution were analyzed using the non-parametric test to identify differences between the two groups. Kruskal–Wallis test followed by dunn.test function (in the dunn.test package) were used for multiple comparison testing. Linear discriminant analysis combined with effect size (LEfSe) algorithm measurements was used to identify biomarkers unique to each group based on the abundance values (25). The cutoff of LDA score in the LEfSe analysis is 2.0. Principal coordinate analysis (PCoA) of data on the gut microbiota was conducted according to the Bray–Curtis distance, and significant differences between the groups were determined using the adonis2 function of vegan 2.5-7. PICRUSt data were analyzed using Statistical Analysis of Metagenomic Profiles (STAMP, version 2.1.3) (26). The Mantel test (23) (mantel function in vegan 2.5-7) was used to test the correlation between the gut microbiota at T0 and T1 according to the Bray–Curtis distance, and the reported mantel *r* and *p*-values are based on 999 permutations. All graphs were generated using the ggplot2 package in R (27). Statistical analyses were performed using R version 4.2, and *p*-values < 0.05 were considered statistically significant.

### Nucleotide sequence accession numbers

The sequence data used in this article have been deposited in the NCBI database (ac-cession number, SRA: PRJNA899929).

## Results

### Baseline characteristics of the subjects

One hundred subjects were included in this study, and four and two subjects in the CTL and LRa05 groups, respectively, dropped out due to restrictions pertaining to COVID-19 (Figure 1). Table 1 illustrates that there were no significant differences in age, sex, blood pressure, and BMI between the two groups at baseline. The metadata of all volunteers were provided in Supplementary Table 1.

**TABLE 1:** Baseline characteristics of the study subjects.

	CTL_0 *N* = 46	LRa05_0 *N* = 48	*P*-value
Age (Year)	23.0 ± 2.4	22.6 ± 1.6	0.838
Gender (Female)	31 (67.4%)	31 (64.6%)	1.000
Systolic blood pressure (mmHg)	111.5 ± 12.2	111.6 ± 10.5	1.000
Diastolic blood pressure (mmHg)	75.4 ± 9.7	76.8 ± 6.7	0.814
Body mass index	22.6 ± 3.8	24.1 ± 4.4	0.305

The *t*-test was used to compare the groups in terms of age, systolic blood pressure, diastolic blood pressure, and body mass index. The chi-square test was used to compare the groups by gender. CTL0 and LRa05 represent the statuses of the CTL and LRa05 groups at baseline (T0), respectively.

### Changes in physical indicators from before to after the intervention

As shown in Table 2, no significant changes in BMI, body fat percentage, and BSFS results were observed in either the CTL or the LRa05 group. This outcome demonstrates the safety of LRa05 supplementation.

**TABLE 2:** Changes in the subjects’ body mass index values, body fat percentages, and Bristol Stool Form Scale results from before to after the intervention.

	CTL_0 *N* = 46	CTL_1 *N* = 46	LRa05_0 *N* = 48	LRa05_1 *N* = 48	*P*-value
Body mass index	22.6 ± 3.8	22.5 ± 3.6	24.1 ± 4.4	24.0 ± 4.3	0.103
Body fat	28.5 ± 7.5	27.9 ± 7.7	31.6 ± 7.6	30.1 ± 7.4	0.114
Gastrointestinal tract score^#^	0 (0.0%)	0 (0.0%)	2 (4.2%)	0 (0.0%)	0.246

^#^CTL0 and LRa050 represent the statuses of the CTL and LRa05 groups at baseline (T0), respectively. CTL-1 and LRa05-1 represent the statuses of the CTL and LRa05 groups after the 4-week intervention (T1), respectively.

### Effects of the intervention on gut microbiota diversity

**FIGURE 3:**
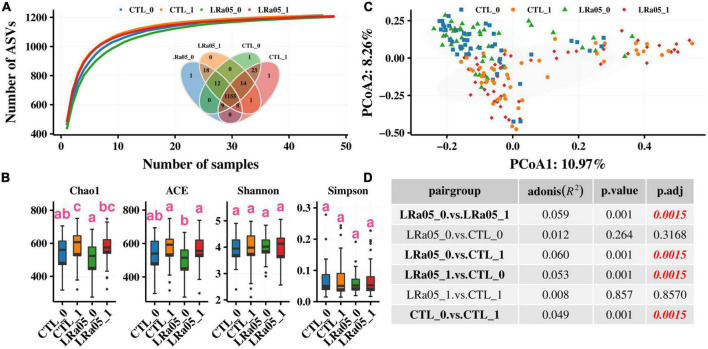
Changes in alpha and beta diversity in the gut microbiota of subjects from before to after the intervention. **(A)** Species accumulation curve. The Venn diagram shows the distribution of species across the CTL and LRa05 groups. **(B)** Changes in alpha diversity from before to after the intervention. **(C)** Changes in beta diversity from before to after the intervention. Principal component analysis based on the Bray–Curtis distance at different consumption stages and in different groups. Each point represents the gut microbiota composition of one subject. **(D)** Comparison of the significance of beta diversity between the groups.

The species accumulation curves (Figure 3A) show successive increases in species in each group, indicating that saturation had been reached in species accumulation, and additional samples could not provide new records. In addition, the Venn diagram in Figure 3A indicates 1,155 species that were common between the CTL and LRa05 groups, accounting for 93.8% of the 1,232 found species. This result suggests that both the placebo and LRa05 intervention resulted in detectable changes in species diversity in the subjects’ gut microbiota. Further diversity analysis (Figure 3B) revealed that both the placebo and LRa05 intervention led to increased species richness (Chao1 and ACE) in the gut microbiota but did not cause significant changes in community diversity (Shannon and Simpson indices). These findings are consistent with the findings from our species accumulation analysis and Venn diagram.

Although alpha diversity analysis did not reveal significant changes in the gut microbiota with placebo and LRa05 intervention, beta diversity analysis revealed (Figure 3C) that both the placebo and LRa05 intervention had a significant effect on the gut microbiota composition. No significant differences in the gut microbiota were observed between the CTL and LRa05 groups at T0 and T1 (Figure 3D, *p* = 0.317 and *p* = 0.857). This result suggests that changes in the gut microbiota after intervention were mainly caused by maltodextrin. The effect of probiotic supplementation on the gut microbiota of healthy individuals was not significantly different from the effect of the placebo.

**FIGURE 4:**
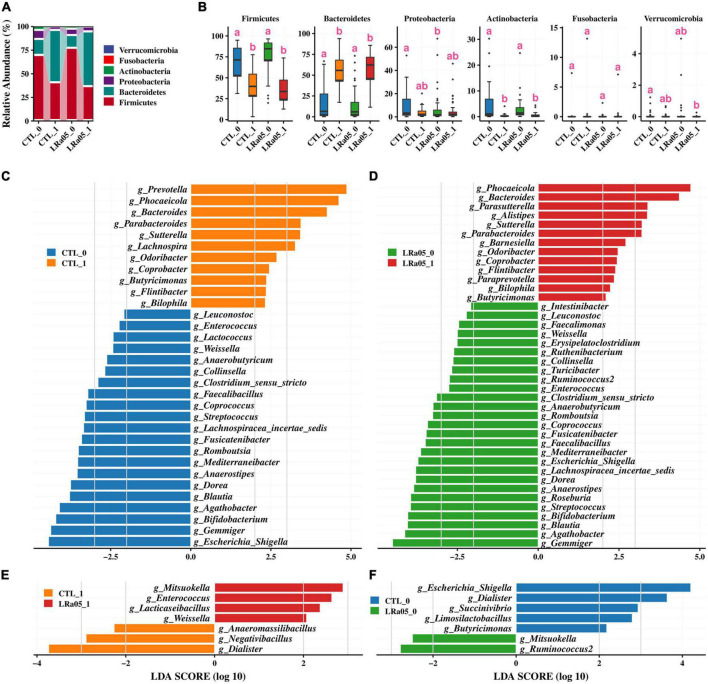
Changes in the gut microbiota composition in the CTL and LRa05 groups from before to after the intervention. **(A)** Distribution of gut microbiota abundance at the phylum level across different groups. **(B)** Changes in abundance at the phylum level from before (T0) to after the intervention (T1). **(C)** Results of LEfSe analysis at the genus level before (T0) and after the intervention (T1) in the CTL group. The cutoff of LDA score in the LEfSe analysis is 2.0. **(D)** Results of LEfSe analysis at the genus level before (T0) and after the intervention (T1) in the LRa05 group. **(E)** Results of LEfSe analysis at the genus level for the CTL and LRa05 groups after the intervention (T1). **(F)** Results of LEfSe analysis at the genus level for the CTL and LRa05 groups at baseline (T0).

At the phylum level, *Firmicutes*, *Bacteroidetes*, *Proteobacteria*, and *Actinobacteria* were the four most predominant bacterial phyla in the subjects’ gut microbiota (Figure 4A). Our analysis of the gut microbial data at T0 and T1 showed no significant phylum-level difference in the gut microbiota between the CTL and LRa05 groups at either time point (Figure 4B), except for *Proteobacteria*. However, both the placebo and LRa05 intervention resulted in significant changes in the gut microbiota composition at T1 relative to T0; these changes mainly involved significant reductions in the relative abundances of *Firmicutes* and *Actinobacteria* and a significant increase in the relative abundance of *Bacteroidetes*.

### Genus-level changes in the gut microbiota and functional analysis

We further investigated changes in the gut microbiota at the genus level using LEfSe analysis. Compared with T0, placebo supplementation resulted in significant increases in the relative abundances of *Prevotella*, *Phocaeicola*, *Bacteroides*, and *Parabacteroides* and significant decreases in the relative abundances of *Escherichia*/*Shigella*, *Gemmiger*, and *Bifidobacterium* at T1 (Figure 4C). LRa05 intervention led to changes in the gut microbiota similar to those observed with the placebo, including significant increases in the relative abundances of *Phocaeicola*, *Bacteroides*, and *Parabacteroides* and significant decreases in the relative abundances of *Escherichia*/*Shigella*, *Gemmiger*, and *Bifidobacterium* at T1 relative to T0 (Figure 4D). Further investigation of the differences between the LRa05 and CTL groups revealed that probiotic intervention led to significant increases in the relative abundances of *Weissella*, *Lacticaseibacillus*, *Enterococcus*, and *Mitsuokella* and significant decreases in the relative abundances of *Dialister*, *Negativibacillus*, and *Anaeromassilibacillus* at T1 (Figure 4E). However, these differences were not observed at T0 (Figure 4F). These results indicate that although the observed changes in the gut microbiota were mainly caused by maltodextrin, the addition of LRa05 led to changes in the relative abundances of some specific microorganisms, such as an increase in *Lacticaseibacillus*. As LRa05 belongs to the genus *Lacticaseibacillus*, this result implies that LRa05 colonization of the gut occurred. Gut microbiota composition information at the family and genus level was also analyzed (Supplementary Figures 1, 2).

**FIGURE 5:**
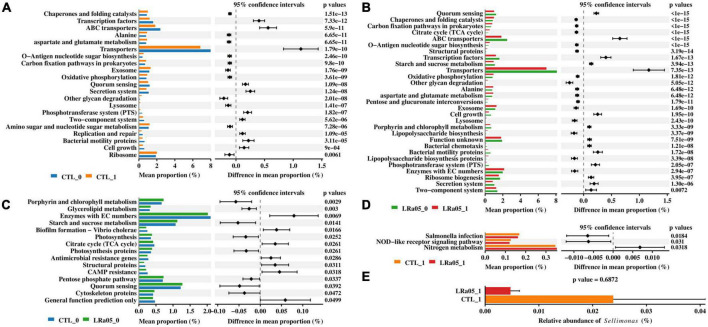
Predictions of functional profiles into KEGG level 3 using PICRUSt and STAMP. The two-sided Welch’s *t*-test was used to compare KEGG functions between **(A)** CTL at T0 (CTL_0) and T1 (CTL_1), **(B)** LRa05 at T0 (LRa05_0) and T1 (LRa05_1), **(C)** CTL_0 and LRa05_0, and **(D)** CLT_1 and LRa05_1 to identify significant differences (corrected *p* < 0.05). The bar plot **(E)** compares the relative abundance of *Sellimonas* between CLT_1 and LRa05_1.

PICRUST analysis showed that the placebo and LRa05 intervention caused similar changes in the function of the microbiota, consistent with the LEfSe analysis results. Compared with T0, both the placebo and LRa05 intervention resulted in the increased abundance of pathways such as chaperones and folding catalysts, alanine, lysosome, and other glycan degradation, as well as significantly decreased abundance of pathways such as ABC transporters, transporters, secretion system, and bacterial motility proteins, at T1 (Figures 5A, B). Only small differences in gut microbiota function were observed between the CTL and LRa05 groups at T0 (Figure 5C), as indicated by smaller differences in mean proportion. At the end of the trial, the abundances of the *Salmonella* infection and NOD-like receptor signaling pathway were reduced and the abundance of the nitrogen metabolism pathway was increased in the LRa05 group compared with the CTL group (Figure 5D). The reduced abundance of the *Salmonella* infection pathway implies the inhibitory potential of LRa05 against harmful bacteria. Therefore, we further compared the abundance of the genus *Sellimonas* at T1 between the CTL and LRa05 groups (Figure 5E). We found that the relative abundance of *Sellimonas* tended to decrease in the probiotic group compared with the placebo group, but this difference was not significant (*p* = 0.6872).

### Correlation analysis of the gut microbiota before and after intervention

**FIGURE 6:**
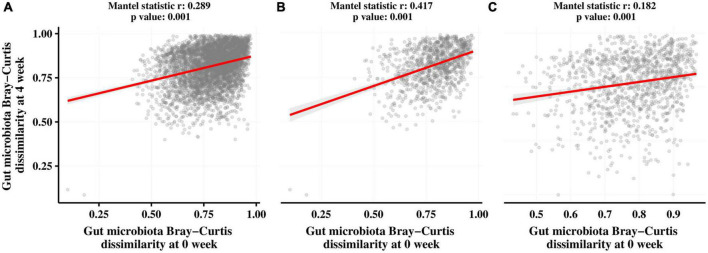
Results of Mantel tests to investigate correlations between the Bray–Curtis distance matrices of the gut microbiota at T0 and T1. **(A)** All subjects. **(B)** Placebo group. **(C)** LRa05 group.

We also assessed the association between the gut microbiota before and after the intervention in both groups using Mantel correlation analysis. As shown in Figure 6, there was a significant correlation (*p* = 0.001) between the gut microbiota at T0 and T1 in all subjects (Figure 5A), the CTL group (Figure 5B), and the LRa05 group (Figure 5C) after the intervention. However, the correlation coefficient was higher in the CTL group (*r* = 0.417) than in the LRa05 group (*r* = 0.182), indicating the effect of LRa05 on the gut microbiota.

## Discussion

LRa05 is an isolate found in infant feces. *In vitro* and *in vivo* studies have demonstrated several properties of LRa05, including acid and bile tolerance, antagonism against enteropathogenic *E. coli*, and immunomodulatory, cholesterol-lowering, and antioxidant effects *in vivo* (17, 18). Therefore, LRa05 is considered a probiotic with potential health benefits. In this study, we analyzed the effect of dietary supplementation with LRa05 on the stability and composition of the gut microbiota of healthy adults using 16S rRNA high-throughput sequencing. We did not observe significant differences in alpha diversity of the gut microbiota between the LRa05 and CTL groups, indicating that this probiotic intervention did not alter the overall stability of the gut microbiota. PCoA visualization further supported the lack of systematic differences in the gut microbiota between the study groups. Our conclusions are consistent with those of a study on *L. rhamnosus* LGG, wherein probiotic intervention did not alter the overall gut microbiota composition in healthy people (28). Additionally, our results showed that although LRa05 supplementation did not significantly affect microbiota stability, it specifically increased the abundance of *Lacticaseibacillus* species, which implies colonization by LRa05 and may also reflect excretion of the ingested strain.

Some studies have indicated that probiotics are conducive to the regulation of the gut microbiota and amelioration of disease (29–31). For individuals with dysbiosis or disruption of the gut microbiota, ingestion of probiotics may modulate the microbiota and reduce gastrointestinal symptoms (32, 33). However, the effects of probiotics are less easily assessed in healthy people than in people with disease due to the lack of an internationally accepted consensus on normal or healthy fecal microbial communities. Studies on the positive effects of probiotics on gut microbiota regulation in healthy individuals have yielded varied results. Clinical studies have shown an increase in the abundance of lactic acid bacteria in healthy adults after the ingestion of *Lactobacillus* strains (7, 9, 34). However, McNulty et al. (35) showed that consumption of yogurt containing five probiotics did not alter the gut microbiota composition in young adults. Several other clinical studies have revealed that probiotic intervention did not cause significant changes in the fecal microbiota composition in terms of alpha and beta diversity compared with a placebo. In addition, several clinical studies have shown that probiotic intervention did not cause significant changes in fecal microbiota composition in terms of alpha diversity and beta diversity compared with placebo (11–15, 28). In a clinical study, probiotic intervention did not significantly affect the alpha diversity of the gut microbiota but had a significant effect on beta diversity (36). Our study involved healthy adult volunteers, namely, young college students whose microbial communities appeared to be well established and balanced.

Despite the lack of consensus regarding a healthy gut microbial composition, the evidence from phylum-level gut microbial gene analyses supports the prioritization of *Firmicutes* and *Bacteroidetes* in healthy individuals (37). Our results are consistent with this prioritization. Additionally, the variance in gut microbiota composition among healthy individuals indicates that the microbiota of each person contains a specific and variable number of bacterial species in addition to the predominant species (38–40). Our results show that maltodextrin significantly modulated the gut microbiota of our healthy subjects, whereas probiotics did not significantly alter the gut microbiota. From an ecological point of view, one bacterial strain would be unlikely to cause fundamental changes to established intestinal communities (41). PICRUSt analysis showed significant changes in gut microbiota function in both the CTL and LRa05 groups at T1 relative to T0. The functional characterization of a healthy gut microbial community remains elusive (42). The effect of probiotics on the gut microbiota composition is only an intermediate result, and the effect on host health should be carefully considered when interpreting it. In addition, we observed the enrichment of *Lacticaseibacillus* in the LRa05 group compared with the CTL group and demonstrated the potential of LRa05 to inhibit *Salmonella* infection, as indicated by the results of our PICRUSt analysis.

In conclusion, we identified a modulatory effect of LRa05 on the gut microbiota *via* high-throughput sequencing analysis. Our results partially confirm and extend previous observations from animal studies and inform hypotheses to support subsequent studies in humans. However, this study has some limitations. First, the analysis is based on a Chinese cohort of young adults within a small age range. Second, our analysis included only two time points and did not consider dynamic changes in the gut microbiota. Third, although probiotics have been reported to enhance immunity, we did not test other blood parameters. Despite these limitations, the changes that we observed in the fecal microbiota composition of healthy adults after LRa05 intervention could provide insight into the mechanisms underlying the effects of probiotics and the fecal microbiota. Furthermore, the use of blood parameters or metabolic profiling and clinical studies of disease cohorts to confirm our findings is necessary to assess the regulatory effects of probiotics on the gut microbiota and the associated effects on human health.

## Conclusion

In summary, both the placebo and LRa05 intervention led to significant changes in the gut microbiota of healthy adults relative to baseline. However, there were no significant differences in alpha and beta diversity between the CTL and LRa05 groups, indicating that the observed changes in the gut microbiota were mainly caused by maltodextrin supplementation. Additionally, a significant increase in the abundance of *Lacticaseibacillus* was found in the LRa05 group compared with the CTL group, implying the potential of LRa05 to colonize the human gut. Overall, our findings suggest the tolerance and colonization potential of LRa05 and, in particular, its potential ability to modulate the gut microbiota and reduce the abundance of harmful bacteria.

## Data availability statement

The datasets presented in this study can be found in online repositories. The names of the repository/repositories and accession number(s) can be found in this article/Supplementary material.

## Ethics statement

The study followed the guidelines of the Declaration of Helsinki, and all procedures involving the human body were approved by the Ethics Committee of Henan University of Technology (No. HautEC202277). The patients/participants provided their written informed consent to participate in this study.

## Author contributions

YY and YW: conceptualization, data curation, formal analysis, funding acquisition, software, and writing–original draft. ZG and YD: conceptualization, investigation, methodology, writing–review and editing, and funding acquisition. FX: project administration, resources, supervision, formal analysis, software, validation, and visualization. JZ: data curation, formal analysis, and software. All authors contributed to the article and approved the submitted version.

## Footnotes

1
https://drive5.com/usearch/

2
https://www.drive5.com/usearch/manual
