# Supplementary material for: Changes in the gut microbiota composition of healthy young volunteers after administration of Lacticaseibacillus rhamnosus LRa05: A placebo-controlled study
Source: Front Nutr. 2023 Mar 14;10:1105694. doi: 10.3389/fnut.2023.1105694 (PMC10043436; doi:10.3389/fnut.2023.1105694)
Supplement: Supplementary file 2 [file Image_1.PDF]

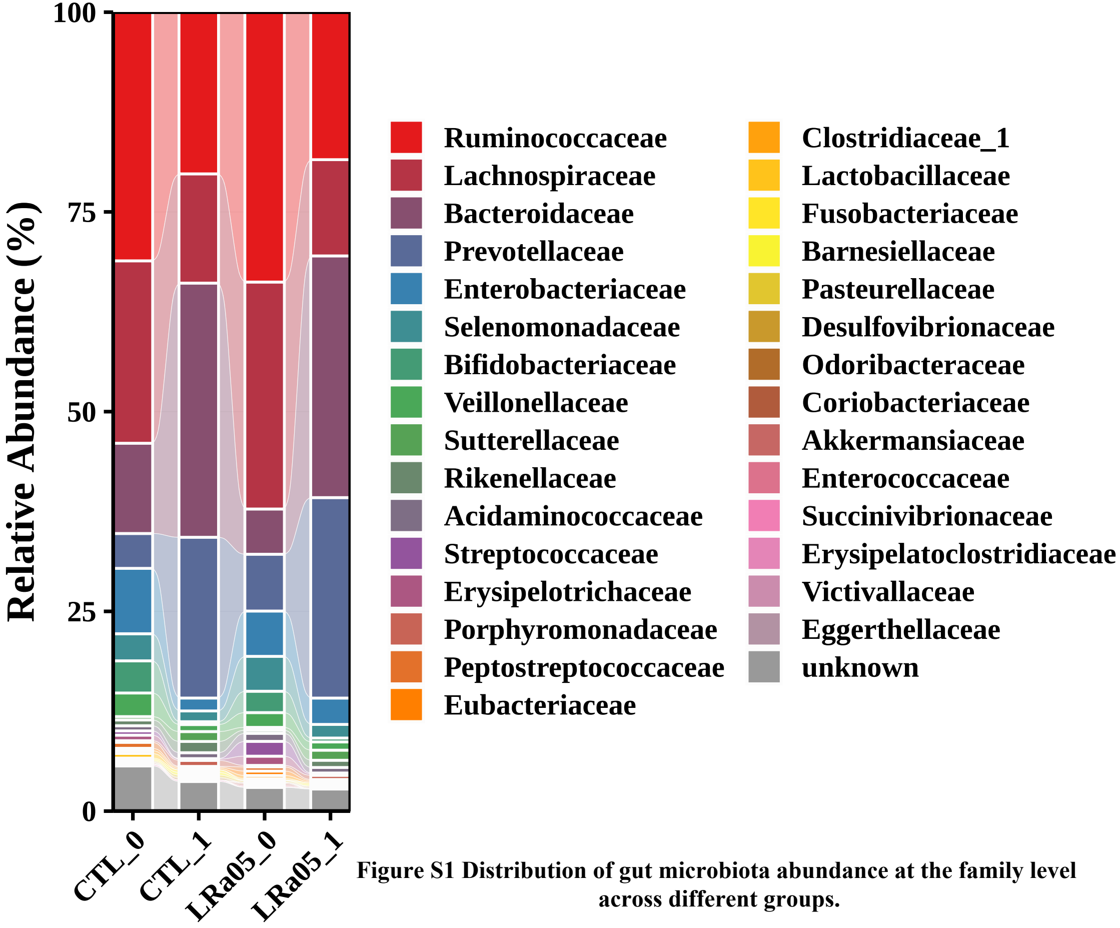

Figure S1 Distribution of gut microbiota abundance at the family level across different groups.
