# Supplementary figures and images for: Changes in the gut microbiota composition of healthy young volunteers after administration of Lacticaseibacillus rhamnosus LRa05: A placebo-controlled study
Source: Front Nutr. 2023 Mar 14;10:1105694. doi: 10.3389/fnut.2023.1105694 (PMC10043436; doi:10.3389/fnut.2023.1105694)

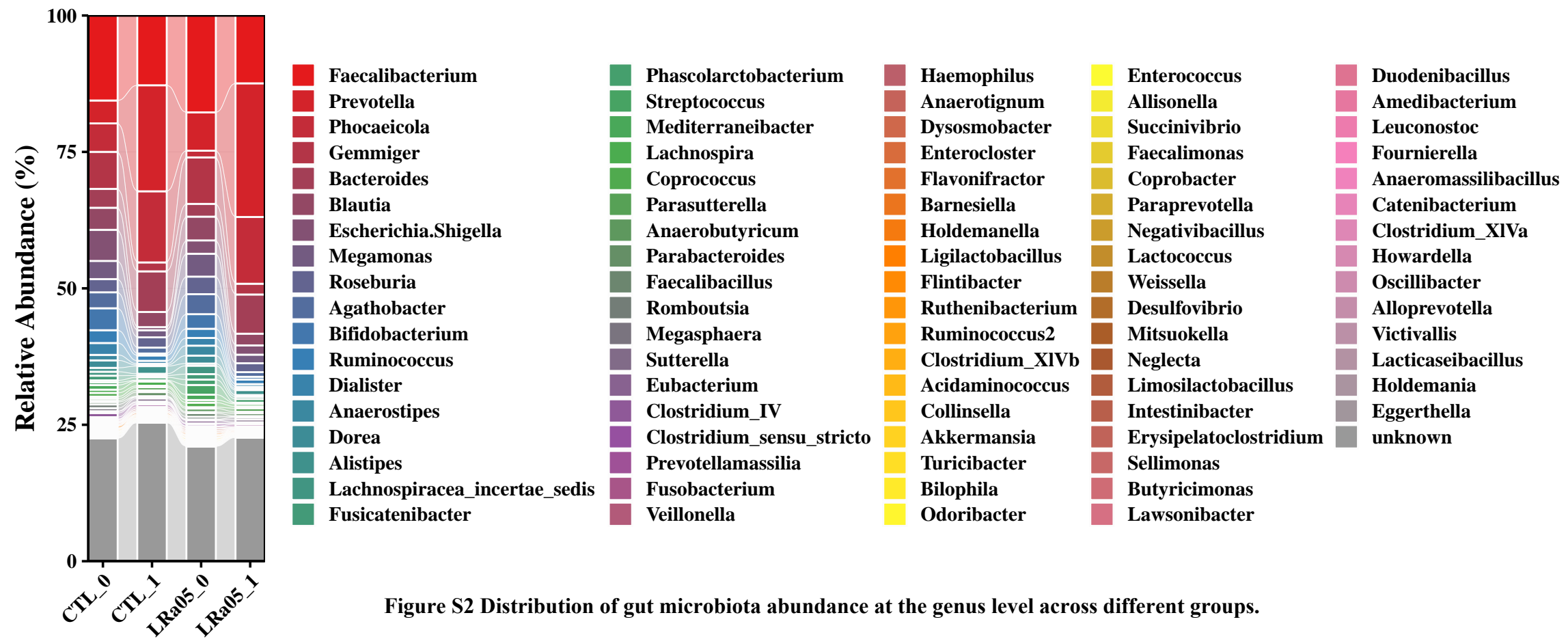

Figure S2 Distribution of gut microbiota abundance at the genus level across different groups.

Supplement: Supplementary file 3 [file Image_2.PDF]
